# Predicting Malignancy and Invasiveness of Pulmonary Subsolid Nodules on CT Images Using Deep Learning

**DOI:** 10.3389/fonc.2021.700158

**Published:** 2021-07-26

**Authors:** Tianle Shen, Runping Hou, Xiaodan Ye, Xiaoyang Li, Junfeng Xiong, Qin Zhang, Chenchen Zhang, Xuwei Cai, Wen Yu, Jun Zhao, Xiaolong Fu

**Affiliations:** ^1^ Department of Radiation Oncology, Shanghai Chest Hospital, Shanghai Jiao Tong University, Shanghai, China; ^2^ School of Biomedical Engineering, Shanghai Jiao Tong University, Shanghai, China; ^3^ Department of Radiology, Shanghai Chest Hospital, Shanghai Jiao Tong University, Shanghai, China

**Keywords:** pulmonary subsolid nodules, computed tomography, diagnosis, computer-aided diagnosis (CAD), deep learning

## Abstract

**Background:**

To develop and validate a deep learning–based model on CT images for the malignancy and invasiveness prediction of pulmonary subsolid nodules (SSNs).

**Materials and Methods:**

This study retrospectively collected patients with pulmonary SSNs treated by surgery in our hospital from 2012 to 2018. Postoperative pathology was used as the diagnostic reference standard. Three-dimensional convolutional neural network (3D CNN) models were constructed using preoperative CT images to predict the malignancy and invasiveness of SSNs. Then, an observer reader study conducted by two thoracic radiologists was used to compare with the CNN model. The diagnostic power of the models was evaluated with receiver operating characteristic curve (ROC) analysis.

**Results:**

A total of 2,614 patients were finally included and randomly divided for training (60.9%), validation (19.1%), and testing (20%). For the benign and malignant classification, the best 3D CNN model achieved a satisfactory AUC of 0.913 (95% CI: 0.885–0.940), sensitivity of 86.1%, and specificity of 83.8% at the optimal decision point, which outperformed all observer readers’ performance (AUC: 0.846±0.031). For pre-invasive and invasive classification of malignant SSNs, the 3D CNN also achieved satisfactory AUC of 0.908 (95% CI: 0.877–0.939), sensitivity of 87.4%, and specificity of 80.8%.

**Conclusion:**

The deep-learning model showed its potential to accurately identify the malignancy and invasiveness of SSNs and thus can help surgeons make treatment decisions.

## Introduction

Lung cancer is one of the most lethal malignancies worldwide (1). Early detection and accurate diagnosis of pulmonary nodules can decrease the mortality of lung cancer (2). According to the content of solid component, pulmonary nodules can be divided into solid nodules and subsolid nodules (SSNs). They have great difference in clinical management due to their different biological characteristics (3).

SSNs are defined as nodular areas of homogeneous or heterogeneous attenuation that did not completely cover the whole lung parenchyma within them, including pure ground-glass nodules (PGGNs) and part-solid nodules (PSNs) (4) (**Supplementary Figure S1**). According to the pathology, SSNs can be further divided into benign and malignant lesions, of which malignant SSNs include pre-invasive (atypical adenomatous hyperplasia, AAH; adenocarcinoma *in situ*, AIS; minimally invasive adenocarcinoma, MIA) and invasive lesions (invasive pulmonary adenocarcinoma, IA) (5). The three categories of SSNs have different biological characteristics and need different clinical management. Benign SSNs include hemorrhage, inflammation, fibrosis, pulmonary alveolar proteinosis, etc. (6), which need almost no intervention but only follow-up. In contrast, malignant SSNs include subtypes of adenocarcinoma, and those malignant pathological types need careful intervention, such as surgical resection and stereotactic body radiation therapy (SBRT) (7). To be specific, receiving systematic lymph node dissection has no statistical significance on improving the prognosis of patients with pre-invasive SSNs (8, 9). The pre-invasive malignant SSNs may just need to be treated with conservative approach (sub-lobectomy or wedge resection) with long-term CT follow-up, while more aggressive surgical treatment (standard lobectomy with extended lymph node dissection) is necessary for patients with invasive (IA) SSNs. Also, the prognosis of different pathological subtypes varies greatly after the corresponding treatment (10, 11). Therefore, accurate classification of SSNs has a great importance for clinical decision-making and prognosis evaluating, especially for thoracic surgeons as it determines the candidates of surgery and the type of lung resection.

Nowadays, the prevalence application of high-resolution CT scanning makes more SSNs be detected at an early stage. However, for those detected SSNs, there exist many difficulties for accurate diagnosis during clinical practice. For example, the synchronous or asynchronous appearance of multiple primary SSNs, the inappropriate location of the SSNs, and the poor physical condition of the patients make it impossible to access each SSN by biopsy. Therefore, CT imaging has become the most important method to help clinicians make the diagnostic decisions of SSNs. As reported, clinicians often make decisions according to some CT morphological features (12, 13). Nevertheless, these morphological features are subjective and qualitative, which often lead to low inter-observer agreement and unsatisfied accuracy (14–16). The inaccurate diagnosis caused by the above limitations have led to undertreatment or overtreatment for patients with SSNs in clinical practice. Therefore, a more objective and quantitative method to accurately distinguish the malignancy and invasiveness of SSNs is urgently needed.

Recently, deep learning has been widely used to analyze medical images on various image modalities (17–20). Previous studies have shown the efficiency of deep learning in pulmonary nodule detection and classification areas (21–23). However, most of these studies are based on solid nodules, and few concentrate on SSNs. Therefore, this study aims to develop and validate a deep learning–based malignancy and invasiveness prediction model in patients with SSNs from the realistic clinical cohort.

## Materials and Methods

### Patients

With approval from the institutional review board, we retrospectively collected patients with pulmonary nodules in Shanghai Chest Hospital from January 1, 2012, to December 31, 2018. The inclusion criteria include the following: (1) Patients received surgical resection of pulmonary nodules in our hospital. (2) Patients received pre-surgery chest CT scanning (thickness ≤5 mm) in our hospital. (3) Subsolid nodules were confirmed in the chest CT. Patients were excluded if (1) post-surgery pathological results were not available; (2) distant metastasis was found in preoperative examinations; (3) other malignant radiological features were present including enlarged hilar nodes, pleural effusion, atelectasis, etc.

### CT Image Acquisition and Nodule Segmentation

Chest CT scans were taken with a 64-detector CT row scanner (Brilliance 64; Philips, Eindhoven, Netherlands). Part of the patients conducted a target thin-section helical CT scan with layer thickness of 1 mm, while the others only had the whole lung scan with a layer thickness of 5 mm.

SSNs were manually segmented by one radiation oncologist (with 5 years of experience in CT interpretation) using the MIM software (version 5.5.1, shown with window level −400 and window width 1,600), then the region of interest (ROI) was confirmed by one radiologist (with over 10 years of experience in CT interpretation).

### Image Preprocessing

The image preprocessing procedure are as follows: CT scans were converted into Hounsfield units (HU), then voxel intensity was clipped to [−1,024, 400] and [−160, 240] HU, respectively. Min-Max normalization was used to rescale the image to [0,1]. Linear interpolation was applied to get isotropic volumes with a resolution of 0.5 mm × 0.5 mm × 0.5 mm. Then, an image cube and the corresponding segmentation mask with 64 × 64 × 64 voxels were cropped from the interpolated CT image centered on the tumor. The cropped image cubes were used as the input of our 3D CNN classification model.

### Pathological Information

According to the pathological report, each SSN was given a specific label (benign, AAH, AIS, MIA, IA). For the malignancy classification, patients who had at least one pathologically confirmed malignant SSN (including AAH, AIS, MIA, IA) were regarded as positive samples with label 1, and those without malignant findings were negative samples with label 0. For the invasiveness classification, patients who were pathologically confirmed as AAH, AIS, or MIA were regarded as pre-invasive samples with label 0, while patients confirmed as IA were regarded as invasive samples with label 1.

### Development of the Classification Model

**Figure 1:**
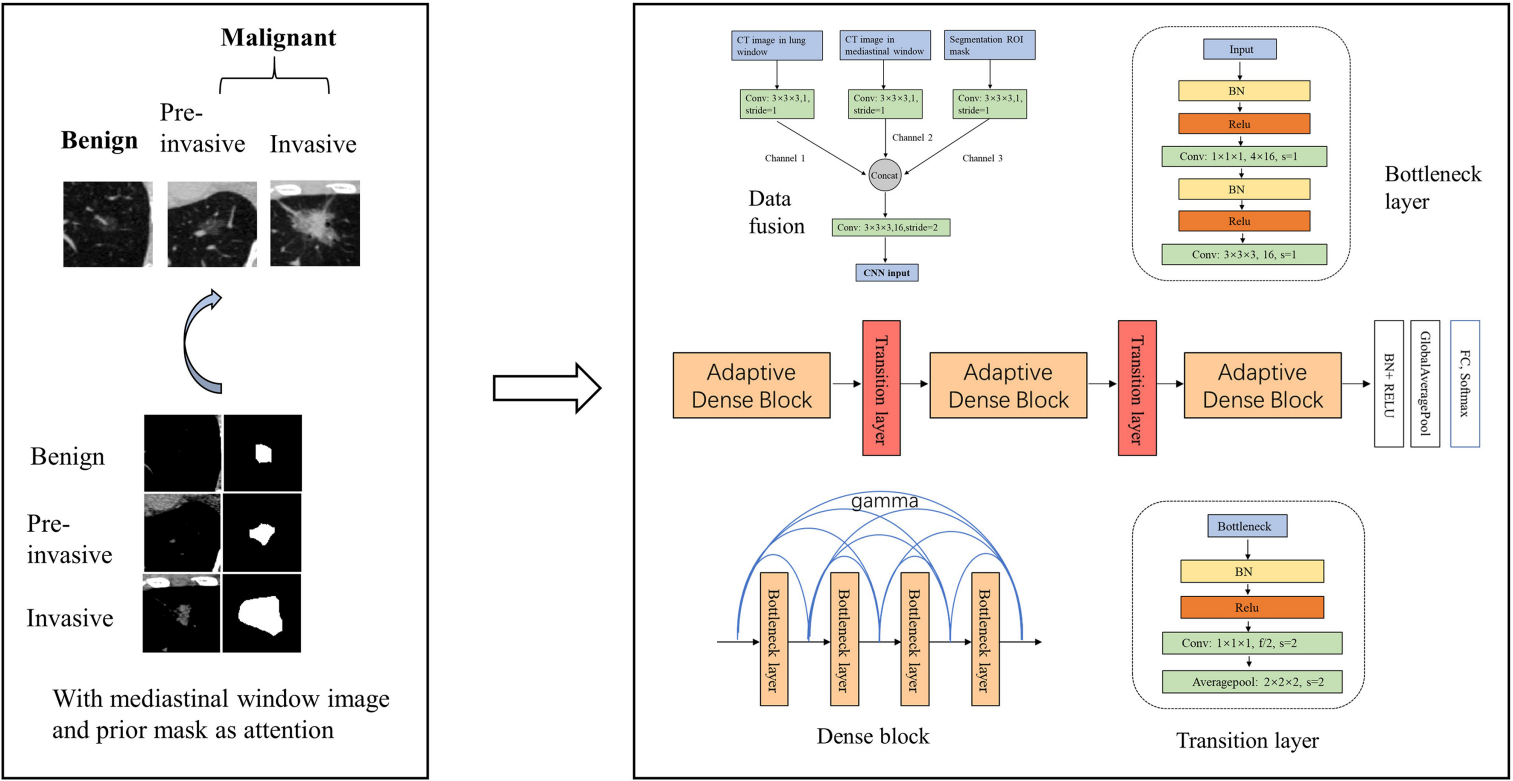
Framework of our model. We developed a 3D CNN model for the malignancy and invasiveness recognition of subsolid pulmonary nodules. The 3D CNN model was based on modified 3D adaptive DenseNet and was improved by incorporating different window images and segmentation mask.

We respectively established a binary classifier to distinguish benign and malignant SSNs and another one to recognize pre-invasive and invasive SSNs. The framework of our models is shown in **Figure 1**.

We totally constructed three models for the malignancy and invasiveness prediction of SSNs, respectively. First, a logistic regression model built with nodule size was used as the baseline clinical model. Second, a 3D CNN model based on modified adaptive DenseNet using the lung window image as input was constructed (AdaDense) (24). The adaptive dense connected structure can effectively reuse the shallow layers’ features by allowing each layer access to feature maps from all of its preceding layers, which makes it easier to get a smooth decision function with better generalization performance. However, as most of the subsolid nodules’ size are small, there exist lots of noisy information from the background in the cropped image patches. Therefore, we considered incorporating the segmentation mask as attention map to help the network focus on regions within the nodule. Moreover, studies have shown that solid portions of SSNs detected by mediastinal window can help distinguish pure ground-glass nodules and part-solid nodules (25, 26), and the proportion of solid components are considered to be related with the malignancy and invasiveness classification (8, 27). Therefore, to take the segmentation mask and solid component factors into account, we finally built another 3D CNN model using the lung window image [HU: (−1,024,400)] incorporated with mediastinal window image [HU: (−160,240)] and mask image as input (AdaDense_M). Then, given the CT image of SSNs, the CNN model output the predicted probability of the SSN being malignancy or invasiveness.

The architecture of the AdaDense_M model can be seen in **Figure 1**, which consists of two parts, data fusion and main structure. For the data fusion part, the CT image patch in different windows and the corresponding segmentation mask were separately convolved by a kernel of 3×3×3 to obtain channels 1, 2, and 3, respectively. Then the three channels were concatenated together and convolved by a 3×3×3 kernel with stride=2 as the input of the main structure. This operation reduced the original feature map of 64×64×64 to the size of 32×32×32. For the main structure part, there were three dense blocks connected by transition layers. Each of the dense block contained four bottleneck structures, and after each bottleneck layer, all feature maps in the previous layers were adaptively concentrated together to realize feature reuse. The bottleneck layer can reduce the number of input feature maps, thereby improving the computational efficiency. The transition layer further compressed parameters by reducing half of the feature maps after dense blocks.

As the sample size was limited, we used data augmentation to avoid overfitting. We did online augmentation including rotations, reflection, and translation. For a given nodule patch and the corresponding mask, they were first translated by one to three voxels in three directions. Then the translated images were randomly rotated by 90, 180, 270, and 360° around the *x*-, *y*-, and *z*-axis. Finally, the rotated images were randomly flipped along the *x*-, *y*-, and *z*-axis.

For the network training, we used cross-entropy function as loss function and Adam optimizer to train the model. Xavier was used to initialize the network. The learning rate was set to 1e-4. Maximum iterative epoch was 1,000. We early stopped the training process when the validation dataset’s performance had no improvement within five epochs. The batch size for each iteration was set to 24. The multiple test method was used to improve the stability of testing performance. Given a test example, the input image patch with different windows and the corresponding mask was randomly generated 10 times to obtain 10 different prediction probabilities, and the final prediction result was computed by averaging all prediction probabilities. The study was implemented with Tensorflow framework on a GeForce GTX 1080Ti GPU.

### Observer Reader Study

To compare the performance of the CNN model with human experts for malignancy prediction, an observer reader study was conducted in the same testing dataset. Two radiologists (with over 10 years of clinical experience) were respectively asked to grade the SSNs based on preoperative CT images. The scores ranged from 0 to 10, and the higher the score was, the more likely they thought the SSN was malignant. The detailed scoring criteria can be found in **Supplementary Figure S2**. The radiologists made their own decisions independently. Also, the radiologists were given access to patients’ demographics and clinical history as auxiliary information.

### Model Evaluation and Statistical Analysis

To evaluate different models’ performance, the receiver operating characteristic curve (ROC) was plotted, and the area under the ROC curve (AUC), sensitivity, and specificity were calculated to evaluate these models’ discrimination ability. Delong test was used to pairwise compare different ROCs. Calibration curve was utilized to assess the calibration ability of the model. Brier score was calculated to quantify the calibration of those models, of which lower values (closer to 0) indicate better calibration. Decision curve analysis was used to determine the clinical usefulness of different models by calculating the net benefit of the constructed models at different threshold probabilities.

Mann-Whitney test was used to compare differences of the mean value of patient’s age and max diameter in different groups. Pearson’s χ2 test was used to compare differences of patients’ gender and location proportion in different groups.

The statistical analysis was conducted with R software (Rproject.org) and python (version 3.7). P-value less than 0.05 was considered as statistically significant difference.

## Results

### Patient Characteristics

From the total of 2,614 patients, 1,791 were malignant and 823 were benign nodules. The number of patients with 1 mm layer thickness was 1,735 (accounting for 66.4%), while the other 879 (33.6%) patients were with scans of 5 mm thickness. The median nodule diameter was 1 cm. All patients’ characteristic statistical information are shown in **Table 1**. Detailed distribution of nodule sizes is shown in **Supplementary Figure S3**. Generally, female patients with larger diameter and location of right upper and left upper lobe were more likely to be malignant. The patients were randomly divided into training (60.9%), validation (19.1%), and testing datasets (20%) for the following analysis. The distribution of different subtypes of SSNs on each dataset is shown in **Table 2**. No significant difference was found among the datasets (**Supplementary Table S1**).

**Table 1:** Clinical characteristic of total patients.

Clinical Characteristics	Total Patients (n=2,614)	Malignant Nodules (n=1,791, 68.5%)	Benign Nodules (n=823, 31.5%)	Statistical Significance (Test Used)
**Gender**				
Male	924 (35.3%)	577 (32.2%)	347 (42.2%)	P<0.0001 (Pearson χ^2^)
Female	1,690 (64.7%)	1,214 (67.8%)	476 (57.8%)	
**Age**				
Median (Range)	57 (15–84)	58 (15–84)	57 (19–81)	P=0.055 (Mann-Whitney)
**Max Diameter (cm)**				
Median (Range)	1.0 (0.2–4.5)	1.1 (0.2–4.5)	0.9 (0.2–4.4)	p<0.0001 (Mann-Whitney)
**Solid Ingredients**				
PGGN^a^	1,768 (67.6%)	1,199 (66.9%)	569 (69.1%)	P=0.286 (Pearson χ^2^)
PSN^b^	846 (32.4%)	592 (33.1%)	254 (30.9%)
**Location**				
Right Upper Lobe	949 (36.3%)	671 (37.5%)	278 (33.8%)	p<0.0001 (Pearson χ^2^)
Right Middle Lobe	198 (7.6%)	117 (6.5%)	81 (9.8%)
Right Lower Lobe	469 (17.9%)	289 (16.1%)	180 (21.9%)
Left Upper Lobe	670 (25.6%)	505 (28.2%)	165 (20.0%)
Left Lower Lobe	328 (12.5%)	209 (11.7%)	119 (14.5%)

a PGGN, Pure ground-glass nodules.

b PSN, Part solid nodules.

**Table 2:** Distribution of SSN subtypes on each dataset.

	Training	Validation	Testing	Total
**Benign**	516	154	154	824
**AAH/AIS**	180	53	64	297
**MIA**	371	118	129	618
**IA**	525	175	175	875

### Performance of the Observer Reader Study

The observer readers’ classification ROC, AUC, sensitivity, and specificity are shown in **Table 3** and **Supplementary Figure S4**. As we can see, one radiologist achieved the best performance with an AUC of 0.877 (95% CI: 0.843–0.911), sensitivity of 95.4%, and specificity of 66.7%, which was significantly better than another radiologist reader with an AUC of 0.815 (95% CI: 0.774–0.856). The difference also indicated the low inter-observer agreement of the malignancy recognition in clinical practice.

**Table 3:** Performance of the observer reader study.

	AUC	Sensitivity	Specificity
**Radiologist1**	0.815	80.8%	76.5%
**Radiologist2**	0.877	95.4%	66.7%

### Performance of the 3D CNN Model for Malignancy Prediction

**Figure 2:**
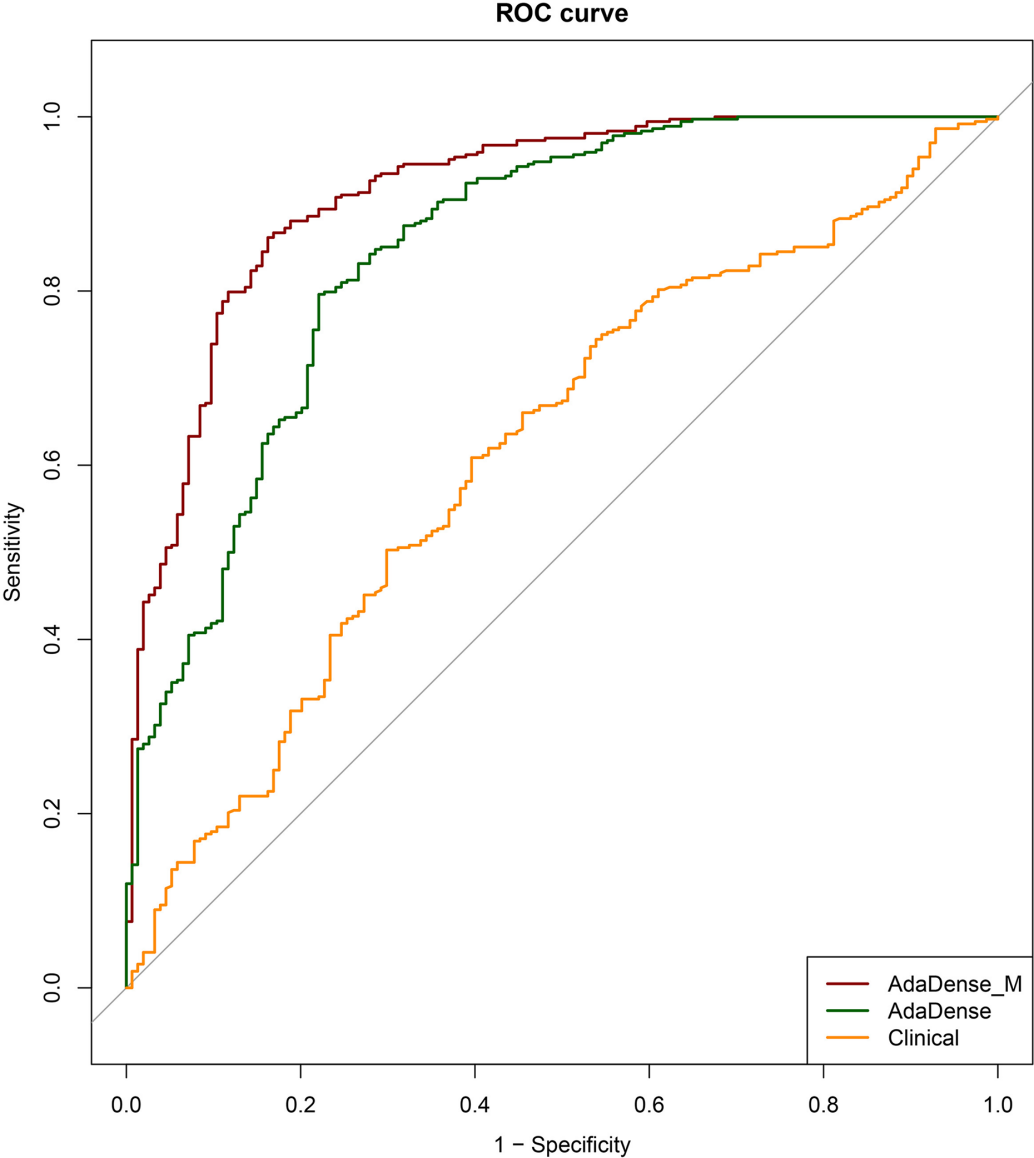
The ROC curves of the CNN models for malignancy prediction. The ROC curves of the AdaDense_M (CNN incorporated with different window images and segmentation mask), AdaDense (CNN only with the lung window image as input), and baseline clinical model (diameter) for malignancy prediction in the testing dataset. The three models’ corresponding AUCs were 0.913, 0.848, and 0.618, respectively. DeLong tests showed that the AdaDense_M performs significantly better than the AdaDense model and the clinical model (p<0.001).

The ROC curves of the 3D CNN models for malignancy classification in the testing dataset are shown in **Figure 2**. As we can see, the best CNN model based on CT images was 3D CNN incorporated with different window images and the segmentation mask (AdaDense_M). The AUC of the best CNN model was 0.913 (95% CI: 0.885–0.940), which was significantly better than the 3D CNN only with the lung window image input (AdaDense) with an AUC of 0.848 (95% CI: 0.810–0.886). Also, the CNN model performed significantly better than clinical features-based model (AUC: 0.618), and adding clinical features to the CNN model yielded no significant improvement (AUC: 0.914, p = 0.489). The sensitivity and specificity of the AdaDense_M model at the optimal decision point were 86.1 and 83.8%. With a sensitivity of 100, 98, and 95%, the percentages of benign nodules that could be correctly identified was 32.5, 47.4, and 63.0%. Also, the Adadense_M model performed better than all the observer readers (AUC: 0.846±0.031).

**Figure 3:**
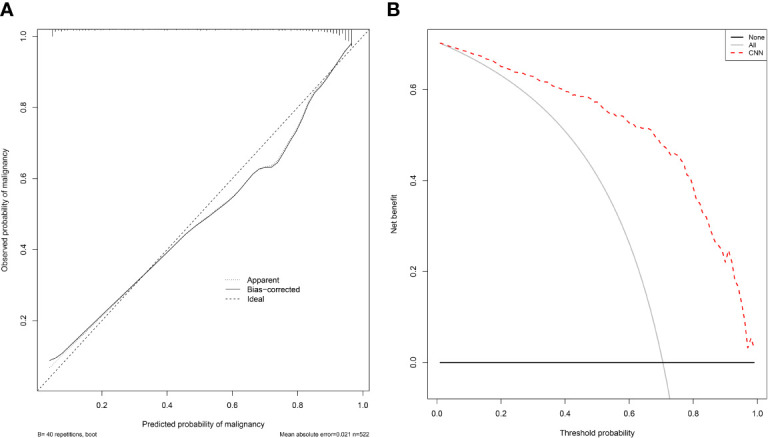
The calibration curve and decision curve of the CNN model for malignancy prediction. **(A)** The calibration curve of the CNN model (AdaDense_M) for malignancy prediction. The diagonal dotted line represents a perfect prediction by an ideal model. **(B)** The decision curve of the CNN model (AdaDense_M) for malignancy prediction. The gray solid line represents the assumption that all patients had malignant nodules. The black solid line represents the assumption that no patients had malignant nodules. The net benefit was calculated by subtracting the proportion of all patients who are false positive from the proportion who are true positive, weighting by the relative harm of a false-positive and a false-negative result.

The calibration curve and decision curve of the CNN model (AdaDense_M) were plotted in **Figure 3**. The Brier score was 0.101, showing satisfactory consistency between the predicted malignant probability and actual observation (**Figure 3A**). Also, the model can bring apparent benefits for the malignancy classification when the threshold was set to 0.01–0.99 compared with the treat-all strategies (perform surgeries in all patients) (**Figure 3B**).

### Performance of the 3D CNN Model for Invasiveness Prediction

**Figure 4:**
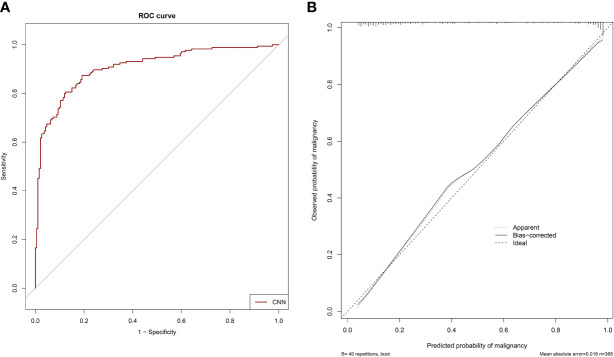
The ROC curve and calibration curve of the CNN model for invasiveness prediction. **(A)** The ROC curve of the CNN model (AdaDense_M) for invasiveness prediction with an AUC of 0.908 in the testing dataset. **(B)** The calibration curve of the CNN model (AdaDense_M) for invasiveness prediction in the testing dataset. The diagonal dotted line represents a perfect prediction by an ideal model.

The ROC curves of the 3D CNN models for invasiveness classification in the testing dataset are shown in **Figure 4A**. The CNN model (AdaDense_M) achieved satisfactory AUC of 0.908 (95% CI: 0.877–0.939), sensitivity of 87.4%, and specificity of 80.8% at the optimal decision point. The confusion matrix is shown in **Table 4**. Calibration curve showed satisfactory consistency between the predicted invasiveness probability and the actual observation with a Brier score of 0.124 (**Figure 4B**).

**Table 4:** Confusion matrix of the CNN model for invasiveness prediction.

CNN prediction			
Ground Truth	Pre-invasive	Invasive	Total
**AAH/AIS**	59	5	64
**MIA**	97	32	129
**IA**	22	153	175

## Discussion

Accurate diagnosis of malignancy and invasiveness of SSNs plays an important role in clinical decision-making, especially for thoracic surgeons. In this study, we developed and validated a novel deep-learning model based on preoperative CT images for accurate classification of SSNs. Moreover, the deep-learning model outperformed radiologists for malignancy prediction.

According to the Fleischner recommendations (3), follow-up CTs are recommended when subsolid nodules are initially detected to differentiate them between transient and persistent. Then, if the nodules are persistent, the management would be determined based on the patient's age, performance status, nodule size, and solid portion size. However, as there exist no national strategy for early-stage lung cancer screening in China, patients with pulmonary nodules may come to the hospital for a variety of reasons. Thus, for Chinese patients in clinical routine, the lesions are usually larger at the first visit, resulting in the risk of diagnosis by dynamic follow-up. Therefore, it is necessary to diagnosis SSNs based on preoperative CT images at a single point. Furthermore, this diagnostic result greatly determines the subsequent treatment strategies in clinical practice. For SSNs that are basically diagnosed as benign, almost no intervention but only follow-up is needed. While for SSNs highly suspicious of malignancy, surgery or SBRT is usually adopted according to the individual condition of patients. More specifically, sub-lobectomy is more appropriate for pre-invasive SSNs, while lobectomy with extended lymph node dissection is more suitable for invasive SSNs. Currently, the inaccurate diagnosis based on radiologists’ subjective judgment may cause overtreatment or undertreatment, which is harmful for the long-term survival of patients. Here, we established a quantitative deep-learning model that can accurately identify the malignancy and invasiveness of SSNs before the operation. This will play an important guiding role in the decision-making of the final surgical resection range, which can avoid unnecessary surgical trauma, reduce the complications of patients, and preserve the lung function to the greatest extent, and at the same time, patients can get radical treatment opportunities.

Considering that CNN has great advantage in automatically extracting deep representative image features, we decided to establish a CNN model for malignancy and invasiveness recognition of SSNs. Our established CNN model incorporated with different window images and segmentation mask (Adadense_M) finally achieved satisfying classification performance. Besides that, we tried to developed a fusion model by combining the CNN model’s prediction result and the best radiologist’s score with logistic regression. The fusion model finally achieved an AUC of 0.956 (95% CI: 0.938–0.975) for malignancy prediction, which was significantly better than the CNN model or radiologist alone. This result means that the CNN model has great potential to help the radiologist make better diagnosis of malignancy of SSNs.

Small sample size was the bottleneck to develop a high-efficacy prediction model for previous studies to distinguish pulmonary SSNs (28–32) (**Table 5**). Our study utilized the largest sample size to date with detailed CT images and pathologic information of SSNs. Compared with models built with qualitative features and radiomics (28–30), our CNN model can automatically learn deep representative features, which have stronger predictive ability than the hand-crafted features. Thus, our CNN model performs significantly better than other radiomics models for malignancy prediction of SSNs. Furthermore, in comparison with models developed with CNN (31, 32), our AdaDense_M model creatively uses the prior segmentation mask and tumor cube in mediastinal window as attention map, which can make the network focus on information within the tumor and its solid components. Results show that the CNN model we built achieved a high AUC value for invasiveness prediction of SSNs among the existing studies.

**Table 5:** Other studies for the classification of pulmonary SSNs.

Author	Sample Size	Method	Task	AUC
**Gon et al. (**28**)**	123 malignant and 59 benign	Radiomics	Benign/Malignant	0.75
**Digumarthy et al. (**29**)**	77 malignant and 31 benign	Radiomics	Benign/Malignant	0.75–0.83
**Yang et al. (**30**)**	920 malignant and 94 benign	Qualitative feature synthesis	Benign/Malignant	0.89
**Gong et al. (**31**)**	828 malignant	CNN	AIS+MIA/IA	0.92
**Zhao et al. (**32**)**	651 malignant	CNN	Pre-invasive/Invasive	0.88

This study also has some limitations. First, we only included patients with pathologically confirmed SSNs who had undergone surgical resection, which results in a selection bias of more malignant patients. If more benign samples can be included, our model would be further improved. Second, there are 33.5% patients who only conducted regular CT scans with the layer thickness of 5 mm. Due to the small size and unique morphology of SSNs, the regular CT scans of SSNs are too blurred to excavate deep features for CNN. More thin-section CT scan data will be collected in the future, and the model performance may be further improved. Moreover, external dataset and prospective cohort are also required to validate the generalization ability of our model.

## Conclusion

We constructed a deep learning–based model to identify the malignancy and invasiveness of pulmonary SSNs based on CT images. The model achieved a satisfactory performance and was proven with potential to guide the selection of surgery candidates and type of lung resection methods.

## Data Availability Statement

The datasets presented in this article are not readily available because the datasets are privately owned by Shanghai Chest Hospital and are not made public. Requests to access the datasets should be directed to XF, xlfu1964@hotmail.com.

## Ethics Statement

The studies involving human participants were reviewed and approved by Shanghai Chest Hospital, Shanghai Jiaotong University. The ethics committee waived the requirement of written informed consent for participation.

## Author Contributions

All authors contributed to the article and approved the submitted version. XF, JZ, TS, and RH contributed to the study concept and design. TS, RH and XL contributed to acquisition of data. RH, TS, XY, XL, JX, QZ, CZ, XC, and WY contributed to analysis and interpretation of data. TS and RH contributed to drafting of the manuscript.

## Funding

This work was supported in part by the Major Research Plan of the National Natural Science Foundation of China (Grant No. 92059206), Shanghai Jiao Tong University Medical Engineering Cross Research Funds (No. YG2017ZD10 and YG2014ZD05), National Key Research and Development Program (No. 2016YFC0905502 and 2016YFC0104608), and National Natural Science Foundation of China (No. 81371634).

## Conflict of Interest

The authors declare that the research was conducted in the absence of any commercial or financial relationships that could be construed as a potential conflict of interest.

## Publisher’s Note

All claims expressed in this article are solely those of the authors and do not necessarily represent those of their affiliated organizations, or those of the publisher, the editors and the reviewers. Any product that may be evaluated in this article, or claim that may be made by its manufacturer, is not guaranteed or endorsed by the publisher.
