# Supplementary material for: Predicting Malignancy and Invasiveness of Pulmonary Subsolid Nodules on CT Images Using Deep Learning
Source: Front Oncol. 2021 Jul 26;11:700158. doi: 10.3389/fonc.2021.700158 (PMC8351466; doi:10.3389/fonc.2021.700158)
Supplement: Supplementary file 1 [file Presentation_1.pdf]

Supplementary Materials

Figures

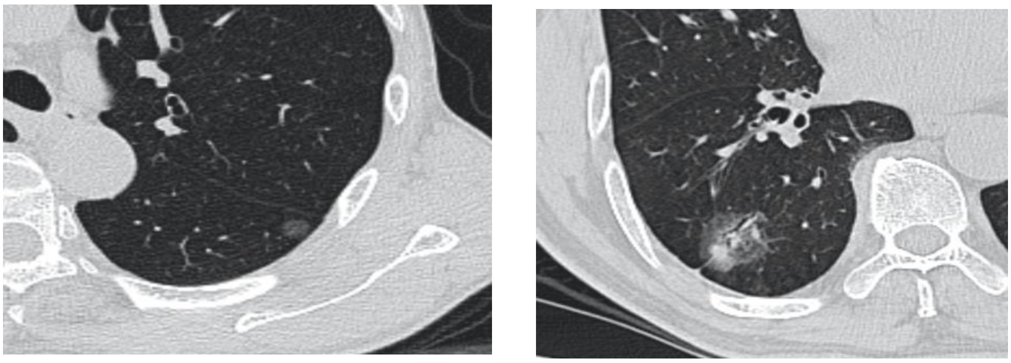

Supplementary figure S1. Typical thoracic CT images of pure ground-glass nodule in left lung and part-solid nodule in right lung.

sure to be benign	highly suspicious of benign	suspicious of benign	difficult to distinguish	suspicious of malignant	highly suspicious of malignant	sure to be malignant
0	2	4	5	6	8	10

Supplementary Figure S2. The scoring criteria of the observer reader study. The scores range from 0 to 10, and the higher the score is, the more likely the observers think the SSN is malignant.

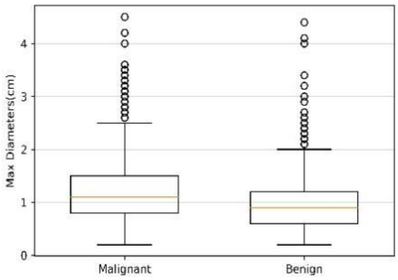

Fig a

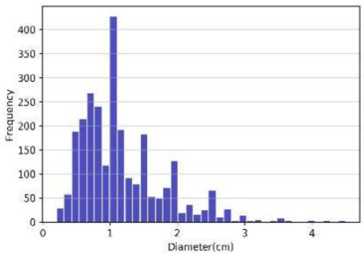

Fig b

Supplementary figure S3. Max diameter distribution of all nodules (Figure a), malignant nodules and benign nodules (Figure b). The median max diameter of total, malignant, and benign nodules was 1.0 cm (0.2-4.5 cm), 1.1 cm (0.2-4.5 cm) and 0.9 cm (0.2-4.4 cm) respectively.

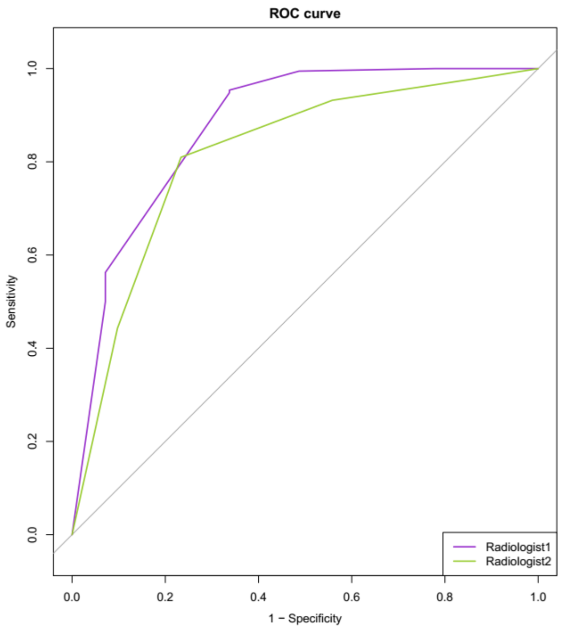

Supplementary figure S4. ROC curves of the observer reader study in the testing dataset. The AUCs of the two radiologists were 0.877 and 0.815, respectively.

Tables

Supplementary Table S1. Clinical characteristics of patients in the training, validation and testing set.

Clinical Characteristics	Total Patients (n=2614)	Training and Validation (n=2092, 80%)	Testing Set (n=522, 20%)	Statistical Significance (Test Used)
Gender				
Male	924 (35.3%)	745 (35.6%)	179 (34.1%)	p=0.608(Pearson χ^2)
Female	1690 (64.7%)	1347 (64.4%)	343 (65.9%)	
Age				
Median (Range)	57 (15-84)	58 (15-84)	57 (23-82)	p=0.268 (Mann-Whitney)
Max Diameter (cm)				
Median (Range)	1.0 (0.2-4.5)	1.0 (0.2-4.5)	1.0 (0.2-3.5)	p=0.422 (Mann-Whitney)
Solid Ingredients				
Pure	1768 (67.6%)	1425 (68.1%)	343 (65.7%)	p=0.318(Pearson χ^2)
Mix	846 (32.4)	667 (31.9%)	179 (34.3%)	
Location				
Right Upper Lobe	949 (36.3%)	759 (36.3%)	190 (36.4%)	p=0.461(Pearson χ^2)
Right Middle Lobe	198 (7.6%)	164 (7.8%)	34 (6.5%)	
Right Lower Lobe	469 (17.9%)	366 (17.5%)	103 (19.7%)	
Left Upper Lobe	670 (25.6%)	546 (26.1%)	124 (23.8%)	
Left Lower Lobe	328 (12.5%)	257 (12.3%)	71 (13.6%)	
Pathologic Type in Details				

Malignant Diseases	Total Malignant Diseases	1791 (68.5%)	1422 (68.0%)	369 (70.7%)	p=0.783 (Pearson χ^2)
	AAH/AIS	298 (16.6%)	233 (16.4%)	65 (17.6%)	
	MIA	618 (34.5%)	489 (34.4%)	129 (35.0%)	
	IA	875 (48.9%)	700 (49.2%)	175 (47.4%)	
